# Mixed T Helper1/T Helper2/T Cytotoxic Profile in Subjects with Chronic Chagas Disease with Hypersensitivity Reactions to Benznidazole

**DOI:** 10.1128/spectrum.01357-22

**Published:** 2022-08-08

**Authors:** Melisa D. Castro Eiro, María A. Natale, María G. Alvarez, Araceli Castro, Débora Seigelshifer, Rodolfo Viotti, Marisa Fernández, Luis Mazzuoccolo, Bruno Lococo, Graciela L. Bertocchi, Gonzalo Cesar, María C. Albareda, María J. Elias, María B. Caputo, Eduardo Gaddi, Jeanette Balbaryski, Carlos A. Vigliano, Susana A. Laucella

**Affiliations:** a Hospital Interzonal General de Agudos Eva Perón, Buenos Aires, Argentina; b Instituto Nacional de Parasitología Dr. Mario Fatala Chaben, Buenos Aires, Argentina; c Instituto de Medicina Traslacional, Trasplante y Bioingeniería (IMeTTyB), Universidad Favaloro-CONICET, Buenos Aires, Argentina; d Hospital Pedro de Elizalde, Buenos Aires, Argentina; University of Arkansas for Medical Sciences

**Keywords:** benznidazole, adverse drug reaction, *Trypanosoma cruzi*

## Abstract

Dermatitis is the most common adverse event during treatment with benznidazole in chronic Chagas disease and is probably mediated by T cells. A set of molecules representative of the different type IV hypersensitivity reactions was evaluated in the circulation and skin biopsies of Trypanosoma cruzi-infected subjects presenting dermatitis during benznidazole administration. Through cytometric bead assays and enzyme-linked immunosorbent assay capture techniques, the serum levels of cytokines, chemokines, proapoptotic molecules, and mediators of the activation and migration of eosinophils and T cells were measured in subjects infected with Trypanosoma cruzi who exhibited skin adverse events (*n* = 22) and compared with those without adverse events (*n* = 37) during benznidazole therapy. Serum levels of interleukin- 5 (IL-5), soluble Fas cell surface death receptor ligand (FAS-L), and interferon γ-induced protein (IP-10) significantly increased at 7 to 30 days posttreatment with benznidazole and decreased thereafter in subjects with dermatitis but not in those without dermatitis. Circulating eotaxin levels were lower in subjects with dermatitis than in those without. Two patterns emerged in the skin biopsies: a T helper 1/T cytotoxic profile and a T helper 2/T cytotoxic profile with the presence of CD4^+^ and CD8^+^ T cells. Increased low-density lipoprotein (LDL), glutamic-oxaloacetic transaminase (GOT), uremia, and T cell activation emerged as risk factors for the development of dermatitis during benznidazole administration. These results support a delayed-type hypersensitivity reaction to benznidazole, involving CD4^+^ and CD8^+^ T cells and eosinophils, and a mixed cytokine profile. This study provides new insights for better management of adverse drug reactions to benznidazole.

**IMPORTANCE** This study identified the risk factors for the development of adverse reactions to benznidazole and identified a set molecule to monitor the appearance of these reactions. This knowledge might improve the safety of benznidazole administration.

## INTRODUCTION

Chagas disease is caused by the intracellular parasite Trypanosoma cruzi and is the main cause of nonischemic cardiomyopathy in Latin America; however, because of the migration flow, it has become a public health concern worldwide (1). Benznidazole and nifurtimox are two drugs approved for the treatment of Chagas disease in the acute and chronic phases of T. cruzi infection. The rate of suspension might reach 30% among adults treated with benznidazole (2–4) mainly because of dermatitis, which is usually expressed as maculopapular exanthema, observed between 7 and 10 days after the initiation of treatment (2).

To correlate the clinical features with the underlying mechanism, hypersensitivity reactions were classified according to the Gell and Coombs classification, from type I to type IV (5–8). Type IV adverse drug reactions (ADRs) are T cell-mediated and are subclassified into four categories according to the profile of cytokines and reactive T cells (9). Although the categories of type IV ADRs might share the induction of exanthema, each category shows a predominant clinical feature with a particular set of immune mediators namely, cytokines, chemokines, granzyme, and perforin (i.e., eczema in type IVa, maculopapular exanthema in type IVb, bullous exanthema in type IVc, and pustular exanthema in type IVd) (9).

Due to the clinical characteristics and onset time of the appearance, dermatitis caused by benznidazole administration is probably a delayed drug hypersensitivity reaction mediated by T cells (10, 11). However, the molecules involved in these hypersensitivity reactions and the presence of T cells in skin biopsies have not been fully characterized.

## RESULTS

### Clinical characteristics of study groups.

All subjects with severe dermatitis and two subjects with moderate dermatitis discontinued benznidazole therapy (Table 1). Five subjects with severe dermatitis received a short course of corticosteroids, and in the remaining subjects with mild or moderate dermatitis, benznidazole was suspended for 48 h, antihistamines were prescribed, and treatment was reintroduced afterward. One of the 11 subjects with severe ADRs showed a drug rash with eosinophilia and systemic symptoms syndrome.

**TABLE 1:** Clinical and epidemiological characteristics of the study population

Group/total (*n*)	Clinical stage at baseline^*a*^		F (*n*)	M (*n*)	Median treatment duration, days (IQR)	Median age at baseline, yr (range)
	G0	G1				
With dermatitis^*b*^						
22						
Mild	7	0	6	1	30	37 (27–50)
Moderate	3	1	2	2	26 (22–30)	44 (35–49)
Severe	9	2	9	2	12 (8–25)	35 (25–49)
Without dermatitis						
37	36	1	27	10	30	38 (23–52)
Uninfected						
24	NA	NA	17	7	NA	44.5 (27-60)

a G0, seropositive individuals with normal echocardiogram and electrocardiogram; G1, seropositive individuals with normal echocardiogram but electrocardiogram abnormalities; F, female; M, male, IQR, interquartile range; NA, not applicable.

b Subjects showing dermatitis during benznidazole administration. Dermatitis was classified as mild, moderate, or severe as explained in Materials and Methods (12).

### Low frequency of benznidazole-reactive cytokine-producing cells in patients with severe dermatitis after benznidazole therapy.

**FIG 1:**
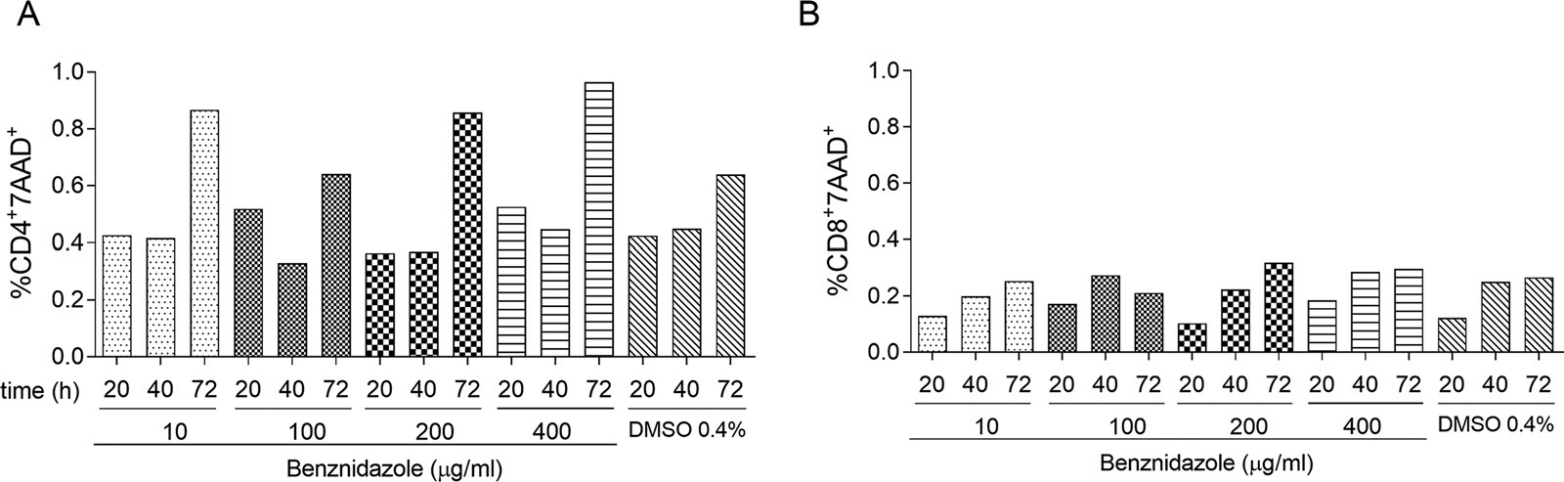
Effect of benznidazole on cell viability. PBMCs were cultured with different concentrations of benznidazole or with the vehicle DMSO for different length of incubation and stained with 7AAD and monoclonal antibodies anti-CD4 (A) and CD8 (B). For the analysis, an area comprising dead lymphocytes was selected according to forward scatter height (FSC-H) and side scatter height (SSC-H) parameters in which the percentages of CD4^+^7AAD^+^ and CD8^+^7AAD^+^ T cells were determined using flow cytometry.

**FIG 2:**
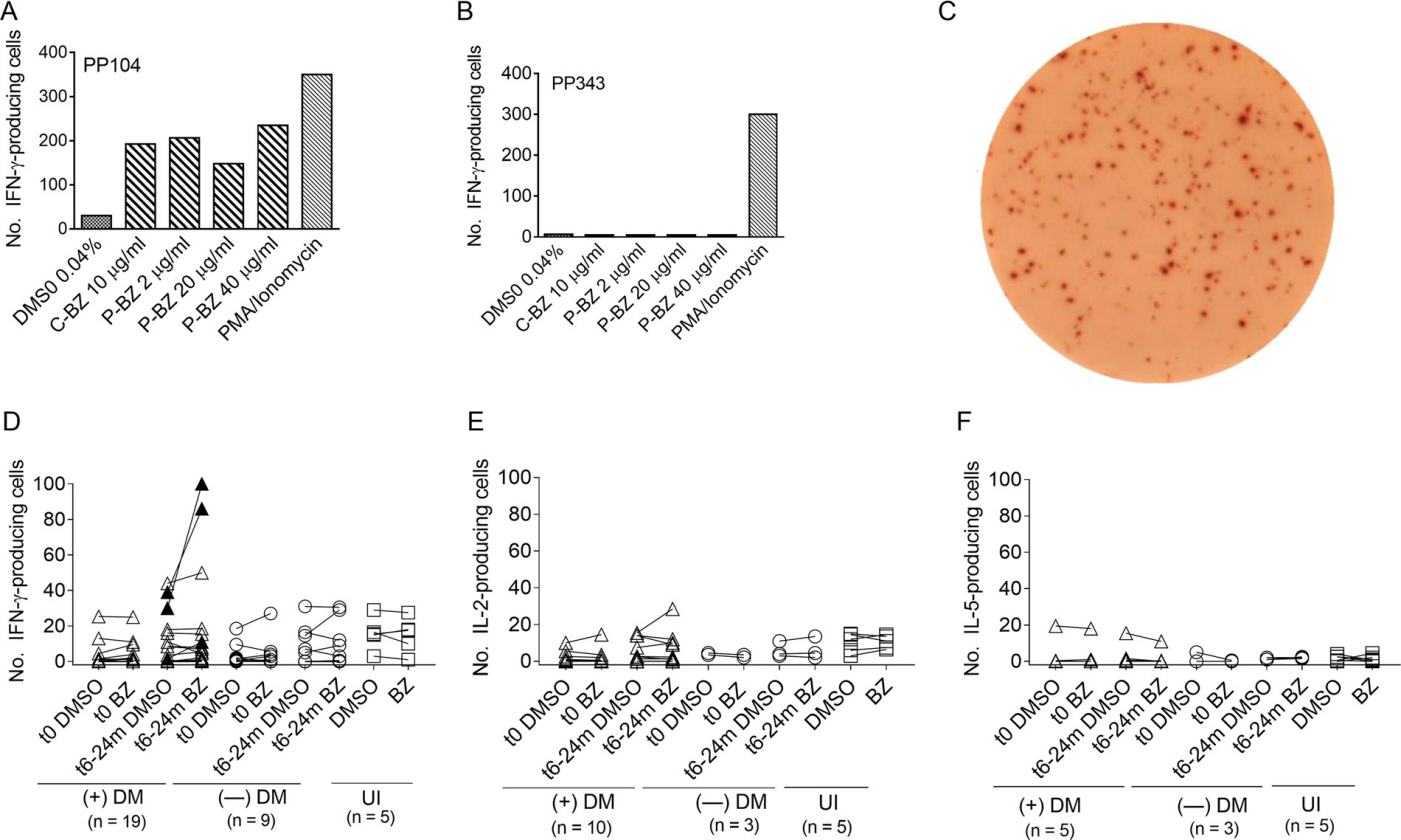
Measurement of benznidazole reactive cells in the circulation of subjects with chronic Chagas disease after benznidazole therapy. For the set-up, PBMCs of one subject with skin ADR (A) and one subject without ADR (B) during benzidazole (Bz) therapy 6 months previously were cultured with different concentrations of benznidazole, the vehicle DMSO, or PMA/ionomycin for 40 h, and the frequency of IFN-γ-producing cells was measured by the ELISPOT technique. Positive ELISPOT responses were defined as described in Materials and Methods. Representative photo of IFN-γ-producing cells by PBMCs of the sample PP104 using 10 μg/mL of pure benznidazole and 40 h of incubation (C). PBMCs were stimulated with 10 μg/mL of pure benznidazole or DMSO and assessed by ELISPOT for IFN-γ (D), IL-2 (E), and IL-5 (F). Each point represents the mean spot number of duplicate wells for each patient. PBMC samples were taken prior to treatment (t0) and at 6 to 24 months (t6-24) after initiation of treatment in T. cruzi-infected subjects with (+DM) or without dermatitis (—DM) during benznidazole therapy and uninfected subjects (UI). Closed triangles indicate positive ELISPOT responses as indicated in materials, and methods. P-BZ, pure benznidazole; C-BZ, commercial benznidazole.

For the set-up, we first established the effect of benznidazole on cell viability and found that the number of dead cells increased on a par with the length of incubation rather than with benznidazole concentrations (Fig. 1A and B). Thereafter, peripheral blood mononuclear cells (PBMCs) isolated from two subjects who had been treated 6 months earlier, (one with dermatitis and one without dermatitis), were cultured in the presence of different concentrations of benznidazole, either pure or derived from pills of the commercial product or with RPMI supplemented with dimethylsulfoxid (DMSO) for 40 hours and analyzed for interferon-γ (IFN-γ) production (Fig. 2A to C). Positive IFN-γ enzyme-linked immunosorbent spot (ELISPOT) responses were recorded in benznidazole-stimulated wells in the range of 2 to 40 μg/mL pure benznidazole in the subject with dermatitis (Fig. 2A and C), whereas no benznidazole-reactive cells were found in the subject without dermatitis (Fig. 2B). We did not find significant differences in the number of IFN-γ-producing cells among the different concentrations assessed or between commercial and pure benznidazole (Fig. 2A and B). Three of the 19 patients with severe dermatitis (i.e., 5 mild, 2 moderate, and 12 subjects with severe dermatitis) had IFN-γ-producing cells reactive to benznidazole between 6 and 24 months after chemotherapy, but not before treatment or in uninfected subjects (UI; Fig. 2D). IL-2- and IL-5-producing cells were also measured, but benznidazole-reactive cells were not found in any of the samples evaluated (Fig. 2E and F).

### Increased serum levels of cytokines and chemokines in benznidazole-induced dermatitis.

**FIG 3:**
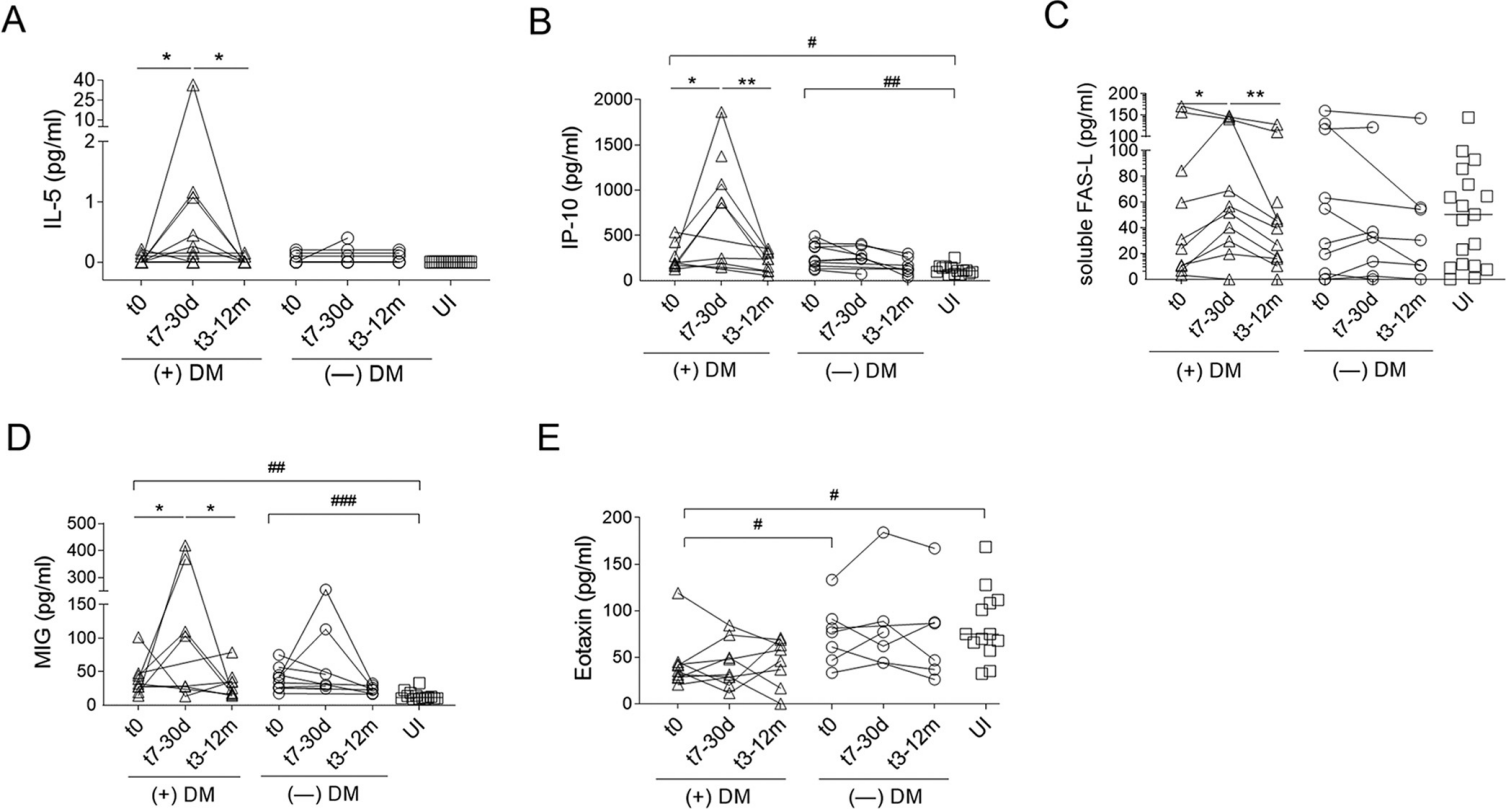
CBA assays for the measurement of cytokines and chemokines in the sera of patients with Chagas disease with adverse skin reactions during treatment with benznidazole. Serum samples were taken prior to treatment (t0), at 7 to 30 days (t17-30d), and at 3 to 12 months (t3-12m) after initiation of treatment in T. cruzi-infected subjects with moderate/severe (+DM; *n* = 11) or without (—DM; *n* = 11) skin reactions during benznidazole therapy and in the uninfected subjects (UI; *n* = 14). Each point represents the concentration values for a single subject for each soluble factor evaluated: IL-5 (A), IP-10 (B), soluble FAS-L (C), MIG (D), and eotaxin (E). *, *P* < 0.05; **, *P* < 0.01, by the Wilcoxon matched-pairs rank sum test. Differences between UI and baseline levels (t0) of T. cruzi-infected subjects are shown as ^#^, *P* < 0.05 and ^##^, *P* < 0.01, and ^###^, *P* < 0.001, as determined by the paired *t* test.

The presence of cytokines and chemokines involved in the differentiation, activation, and recruitment of eosinophils, T cells, and monocytes was evaluated in the sera of subjects infected with T. cruzi with or without benznidazole ADRs (12, 13). The levels of IL-5 (Fig. 3A), IP-10 (Fig. 3B), and soluble FAS-L (Fig. 3C) were significantly increased in the sera of patients with dermatitis, at 7 to 30 days after the initiation of treatment, but not in subjects without dermatitis or uninfected subjects. Although monokine induced by interferon-γ (MIG) levels increased early after treatment in subjects with dermatitis, these levels were also increased in two subjects with no adverse events (Fig. 3D). Baseline IP-10 and MIG levels were increased in subjects with chronic T. cruzi infection compared with the levels in UI, with no difference among subjects with or without ADRs (Fig. 3B and D). Baseline eotaxin levels were decreased in subjects with dermatitis compared with subjects without dermatitis and UI (Fig.  3E), and the levels did not change after treatment. Mixed profiles of Th1 and T cytotoxic and Th2 cells with concomitant increases in one or more cytokines/chemokines of these profiles in the same subject were observed (Fig. S1). There were no significant differences in the levels of granzyme B, IL-13, monocyte chemoattractant protein-1 (MCP-1), regulated on activation, normal T cell expressed and secreted (RANTES), or IL-8 early after the occurrence of adverse events (Fig. S2).

### Increased baseline CD69 expression in CD4^+^ T cells of subjects who underwent skin reactions to benznidazole.

**FIG 4:**
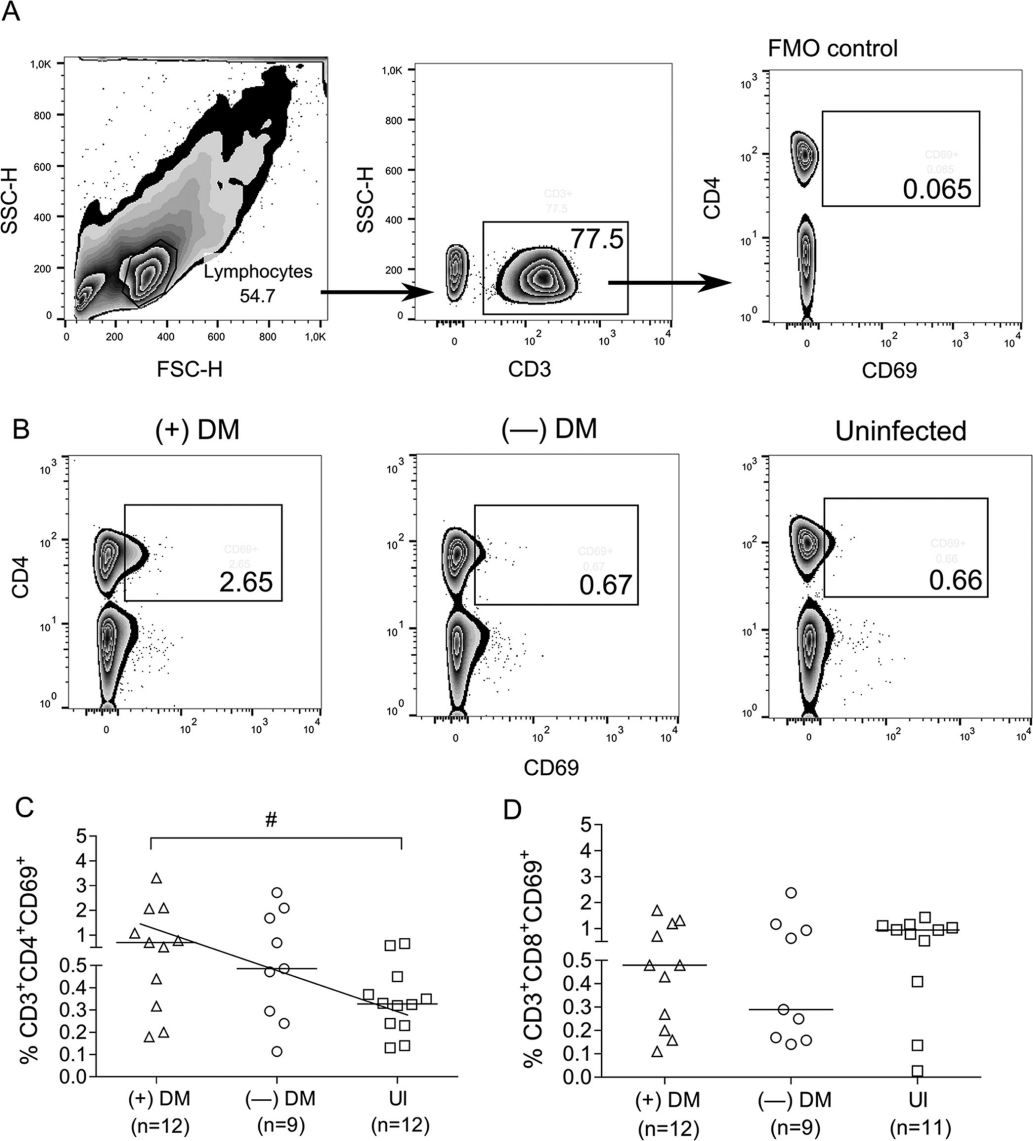
Baseline CD69 expression in subjects with chronic Chagas disease with and without dermatitis following benznidazole therapy. PBMCs were stained with anti-CD3, CD4, CD8, and CD69 monoclonal antibodies and analyzed using flow cytometry. Lymphocytes were gated based on their forward scattering and side scattering parameters, T cells were selected based on CD3 expression, and then CD69^+^ cells were analyzed on CD4^+^ and CD8^+^ T cells according to fluorescence minus one control (FMO) (A). The baseline frequency of CD4^+^ (B and C) and CD8^+^ (D) T cells expressing CD69 was analyzed in PBMCs samples of subjects who subsequently showed dermatitis during benznidazole therapy (i.e., +DM; 2 subjects with mild and 9 subjects with severe DM) and those without adverse events (i.e., —DM) and compared with the levels in uninfected individuals (UI). Data were analyzed by unpaired *t* test or Mann-Whitney test as appropriate. *^#^*, *P* < 0.05 versus the UI group. The diagonal line in C represents a negative trend; slope −0.33, *P* = 0.04.

Because CD69 expression, a marker of recent T cell activation, has been associated with drug hypersensitivity reactions (14), the association between the baseline frequency of CD4^+^ and CD8^+^ T cells expressing CD69 and the subsequent development of ADRs was evaluated. The baseline frequency of CD69^+^CD4^+^ T cells was higher in subjects who presented with skin reactions during benznidazole therapy than that of the uninfected group (Fig. 4A to C), whereas the frequency of CD69^+^CD8^+^ T cells was not different among groups (Fig. 4D).

### CD4^+^ and CD8^+^ T cells in dermatitis biopsies.

**FIG 5:**
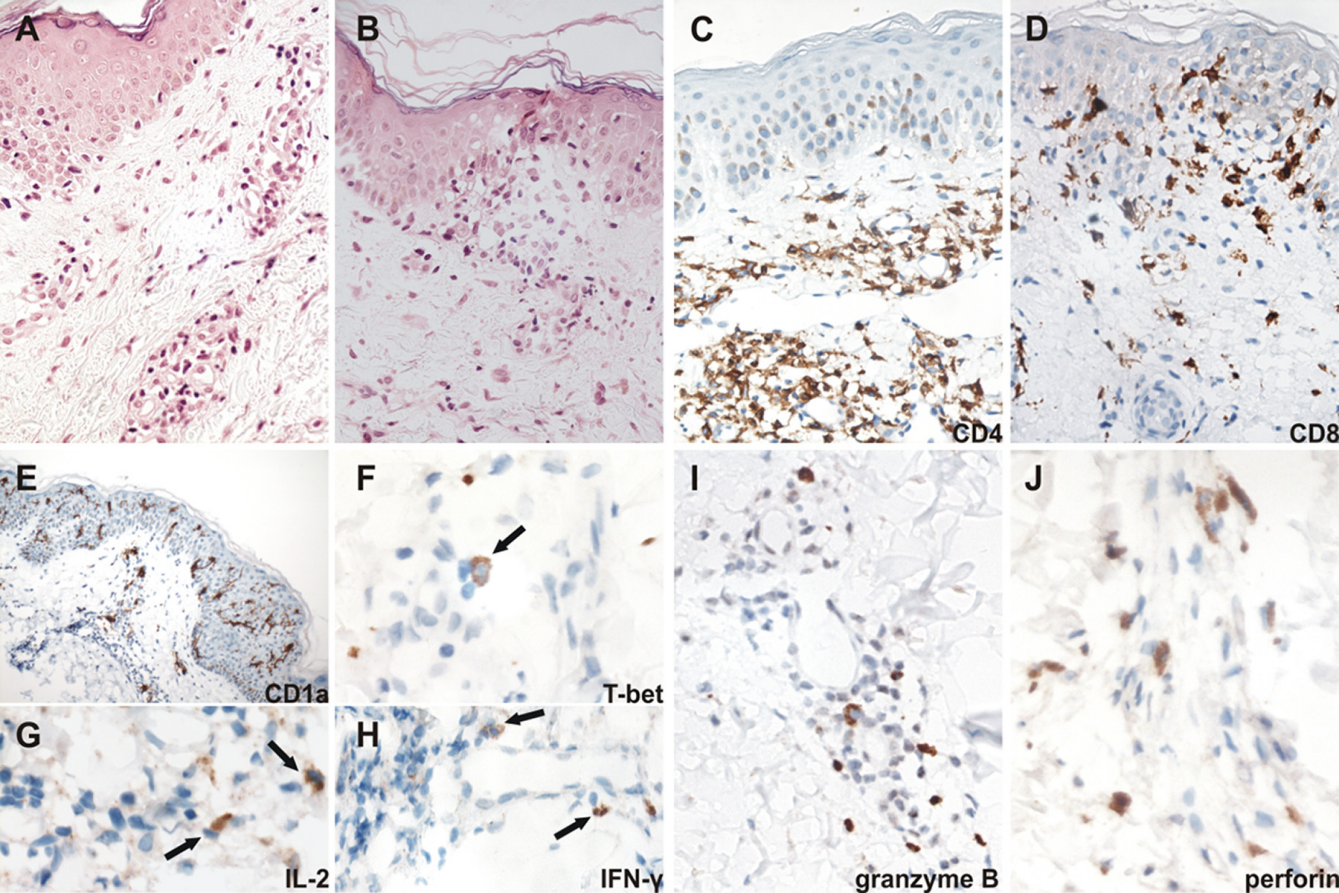
(A) Skin biopsy of a raised erythematous lesion after benznidazole treatment, from a 49-year-old patient with normal clinical laboratory, no eosinophilia, increased transaminase, or leukopenia. H&E, original magnification, ×400. (B) Skin biopsy of the same patient showing inflammatory infiltrate of the epidermis. Hematoxylin and eosin (H&E), ×400. (C) Moderate inflammatory infiltrate in the superficial dermis with a predominance of CD4^+^ T cells. Immunohistochemistry (IHC), original magnification, ×400. (D) The majority of T cells in the epidermis inflammatory infiltrate expressed CD8 (×400). (E) Expression of CD1a in the epidermis and the dermis of the skin lesion (×400). (F) T-bet (arrow) in the inflammatory infiltrate in the dermis (×1,000). (G) IL-2 (arrows) in the dermis inflammatory infiltrate (×1,000). (H) IFN-γ (arrows) in the dermis inflammatory infiltrate (×1,000). (I) Granzyme B in the perivascular inflammatory infiltrate (×400). (J) Perforin in the perivascular inflammatory infiltrate (×1,000).

**FIG 6:**
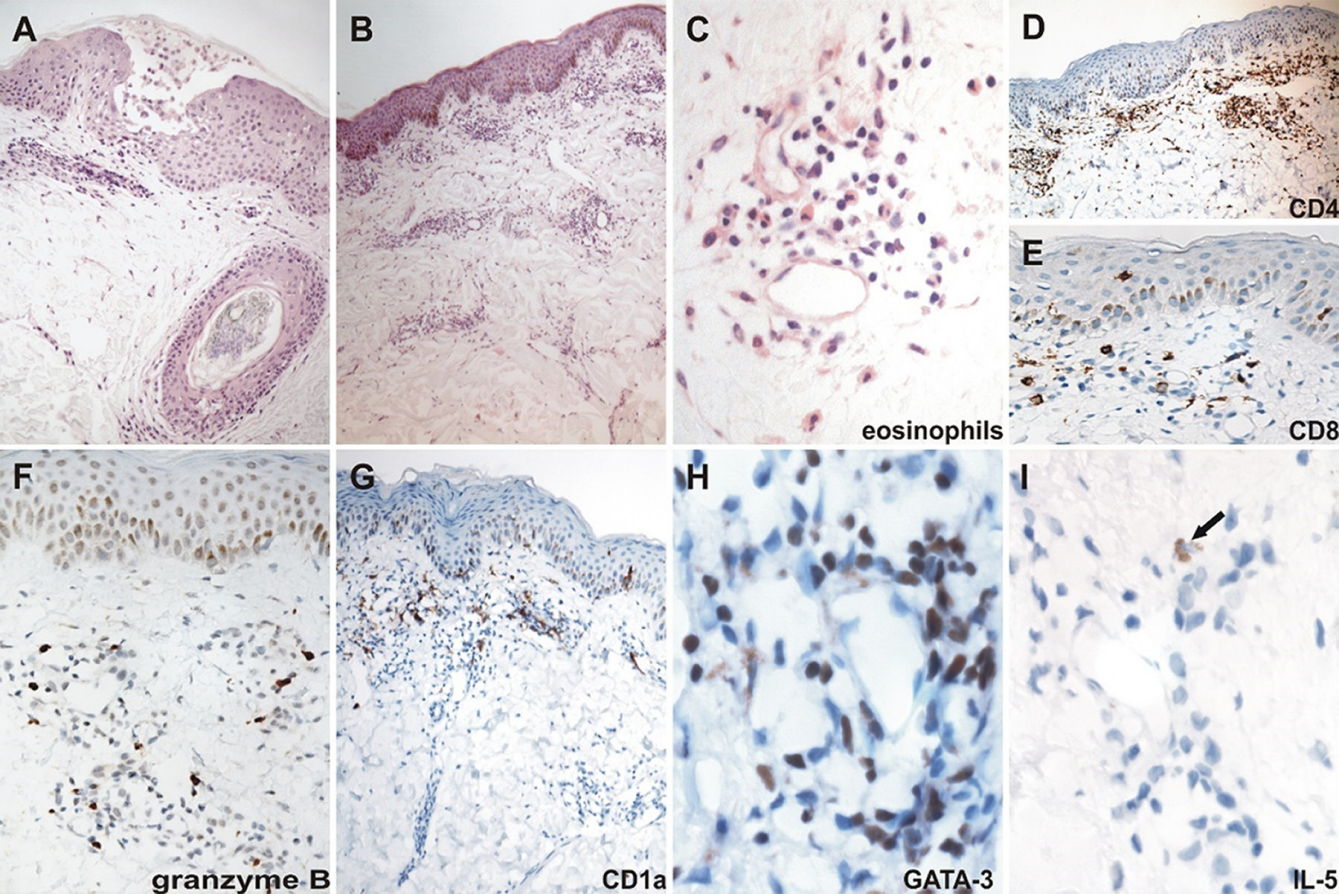
(A) Skin biopsy of generalized maculopapular and pustular exanthema after benznidazole treatment in a man of 29-year-old with peripheral eosinophilia. (H&E, original magnification, ×200). (B) Skin biopsy specimen of a 49-year-old woman being diagnosed with extensive maculopapular cutaneous rash after benznidazole treatment. Sections show superficial and mid-perivascular infiltrates without epidermal alteration. (H&E, ×100). (C) Inflammatory infiltrate in the interstitial and perivascular dermis with a predominance of lymphocytes and eosinophils. (H&E, ×400). (D) Moderate inflammatory infiltrate in the superficial dermis with a predominance of CD4 T cells (IHC original magnification, ×400). (E) Inflammatory infiltrate in the superficial dermis and epidermis, with scarce CD8 intraepidermal T cells along the basal layer and at the lower half of the epidermis (×400). (F) Granzyme B in the perivascular inflammatory infiltrate (×400). (G) Isolated CD1a^+^ cells in the epidermis and with predominantly perivascular distribution in the dermis superficial of the skin lesion (×400). (H) GATA-3 in the perivascular inflammatory infiltrate (×1,000). (I) IL-5 (arrow) in the perivascular infiltrates (×1,000).

In the four biopsies analyzed, perivascular inflammatory infiltrates (Fig. 5A and B and Fig. 6A to C) with the concomitant presence of CD4^+^ and CD8^+^ T cells were observed (Fig. 5C and D and Fig. 6D and E). Representative markers of T helper/cytotoxic 1 and 2 cell profiles were analyzed in four skin biopsies. A mixed Th1-cytotoxic (Fig. 5) profile was associated with normal peripheral levels of eosinophils and comprised the expression of T-box transcription factor (T-bet) (Fig. 5F), IL-2 (Fig. 5G), IFN-γ (Fig. 5H), granzyme B (Fig. 5I), and perforin (Fig. 5J), but with the absence of IL-5. The majority (>90%) of T cells in the epidermis expressed CD8 (Fig. 5D), and few CD4^+^ T cells were found (data not shown). Extensive expression of dendritic cell marker CD1a^+^ was also observed in the epidermis (Fig. 5E). By contrast, T cells in the dermis had both CD4 and CD8 phenotypes, although the former predominated. Notably, increased serum levels of FAS-L were found in two subjects with predominant CD8^+^T cells in their skin biopsies.

In another case, a Th2- cytotoxic (Fig. 6A and B) profile was associated with increased eosinophil numbers (Fig. 6C) in skin tissues and in the circulation and was characterized by the expression of GATA-3 (Fig. 6H) and IL-5 (Fig. 6I), but with the absence of T-bet and IFN-γ. Granzyme B expression was also observed in the perivascular inflammatory infiltrate of the dermis (Fig. 6F). Dermatitis was located in the superficial dermis without the involvement of the epidermis, with CD1a^+^ cells mainly located around the blood vessels (Fig. 6G), and CD8^+^ T cells were only occasionally observed in the epidermis (Fig. 6E). In agreement with a Th2-biased profile, this subject showed increased IgE levels at the time of adverse reactions.

### Relationships of serum biochemical and hematologic biomarkers with appearance of dermatitis during benznidazole administration.

Univariate analysis of a set of biochemical and hematologic biomarkers that assigned the appearance of benznidazole-induced adverse effects as the outcome was evaluated. LDL GOT and uremia levels were higher in subjects who subsequently experienced adverse reactions to benznidazole than in those who did not (Table 2). High LDL levels appeared as an independent risk factor for ADRs during benznidazole administration (*P* = 0.041, odds ratio = 1.02, and 95% CI = 1.05). The percentage of subjects with abnormal LDL levels was also higher (*P* = 0.028) among subjects with dermatitis [i.e., 9/22 (41%)] than in those without dermatitis [i.e., 5/36 (14%)] during benznidazole administration.

**TABLE 2:** Baseline biochemical and hematologic markers in chronic Chagas disease subjects with or without benznidazole-induced dermatitis

Variable (unit)	Benznidazole-induced adverse effects^*a*^			Reference values
	Yes (*n* = 22)	No (*n* = 37)	*P* value^*b*^	
Age (yr, range)	36 (25–50)	38 (23–52)	0.93	NA
Sex (no. female/no. male)	11/11	27/10	0.27	NA
Arterial hypertension (no. subjects/total evaluated, %)	32	11	0.29	120–80 mm Hg
IgE (UI/mL)	76 (1–764)	86 (4–664)	0.58	0.1–100
Leukocytes (total/mL)	7,550 (5,400–9,900)	6,900 (4,500–9,900)	0.24	4,500–10,000
Hemoglobin (g/dL)	14 (12–16)	13 (11–16)	0.08	13.3–16
Platelets (no. ×10^3^/mL)	179 (246–338)	239 (159–311)	0.40	150–450
Neutrophils (no./μL)	3,95 (2.6–7.2)	4 (2.3–7.1)	0.88	2,100–6,200
Eosinophils (no./μL)	0.26 (0–1.02)	0.16 (0–0.74)	0.08	0–500
Basophils (no./μL)	0.05 (0–0.13)	0.05 (0–0.13)	0.69	0–200
Lymphocytes (no./μL)	2.35 (1.3–4.5)	2 (1.2–3.5)	0.27	1,300–3,500
Monocytes (no./μL)	0.46 (0.17–1.03)	0.45 (0–0.95)	0.39	30–1,000
Creatinine (mg/dL)	0.76 (0.60–1.20)	0.7 (0.4–1.18)	0.24	0.7–1.3
Uremia (g/L)	*0.32 (0.20*–*0.47)*	*0.27 (0.14*–*0.41)*	*0.04*	*0.1*–*0.5*
Cholesterol (mg/dL)	208 (135–264)	179 (112–261)	0.15	100–200
Triglycerides levels (mg/dL)	148 (56–448)	120 (33–529)	0.36	50–150
HDL (mg/dL)	49 (33–79)	43 (27–67)	0.05	30–70
LDL (mg/dL)	*126 (75*–*170)*	*104 (0.84*–*187)*	*0.03*	*0*–*130*
TSH levels (U/mL)	2.16 (1.10–23)	2.68 (0.014–5.91)	0.65	0.27–4.2
FT4 levels (ng/dL)	1.14 (0.91–1.81	1.23 (0.95–2.05)	0.14	0.93–1.7
Uric acid (mg/dL)	4.55 (2.5–7.2)	3.7 (2.2–8)	0.27	2.5–6
GOT levels (IU/L)	*25 (7*–*162)*	*19 (11*–*49)*	*0.04*	*0*–*38*
GPT levels (IU/L)	34 (8–115)	20 (6–80)	0.12	0.41
Alkaline phosphatase (IU/mL)	189 (0–331)	186 (72–438)	0.53	65–300
Glucose (g/L)	0.93 (0.77–119)	0.91 (0.7–110)	0.64	0.7–1.1

a Data for continuous variables are shown as medians and ranges.

b For univariate analysis, two-sample *t* test, Wilcoxon rank sum test and the Fisher exact test were used, as appropriate. Italics indicate *P* values lower or equal to 0.05. HDL, high-density lipoprotein; LDL, low-density lipoprotein; TSH, thyroid-stimulating hormone; FT4, free thyroxine; GOT, glutamic-oxaloacetic transaminase; GPT, glutamic-pyruvic transaminase.

## DISCUSSION

Recent findings suggest that cutaneous adverse events during benznidazole administration are produced by a nonimmediate hypersensitivity reaction mediated by T cells (10, 11, 15); thus, we evaluated a set of molecules representative of the different type IV reactions in sera and skin biopsies to identify the mediators participating in hypersensitivity to benznidazole. A mixed simultaneous profile of type IVa, type IVb, and type IVc, involving Th1, Th2, and T cytotoxic mediators, was found in the circulation of individual subjects with chronic Chagas disease showing ADRs to benznidazole. Although the profile in skin biopsies showed the presence of CD4^+^ and CD8^+^ T cells with cytotoxic activity, the profile was skewed toward a Th1 or Th2 phenotype.

Increased serum levels of soluble FAS-L, a mediator of the process of cell death (16), are a common feature in subjects with moderate and severe maculopapular exanthema without the development of bullous eruptions (i.e., Stevens-Johnson syndrome and toxic epidermal necrolysis) after benznidazole administration. The involvement of FAS-L, granzyme B, and perforin in type Vc hypersensitivity reactions, in which keratinocytes are killed, has been well established (17, 18).

Supporting the role of CD8^+^T cells in benznidazole-induced skin ADRs, the presence of intraepidermal CD8^+^ T cells and the expression of granzyme B and perforin were found in skin biopsies. The cytotoxic profile in sera was associated with increased IL-5 levels, Th1-induced chemokines, or both. IL-5, a cytokine of the Th2 profile found in type IVb ADRs (19), is involved in the priming of eosinophils in the peripheral blood, resulting in increased migratory responses and adhesiveness of these cells. Instead, eotaxin participates in the chemotaxis of eosinophils into the inflammatory site (20). Since eotaxin binds to the intracellular matrix via adhesion to proteoglycans to exert its chemotactic activity (20), it is plausible that eotaxin is sequestered in tissues, accounting for the low levels found in the circulation of subjects with ADRs. Accordingly, many eosinophils were found in one skin biopsy in which IL-5 expression was detected along with the presence of CD4^+^ and a low number of CD8^+^ T cells, and five of the eight subjects showing increased serum IL-5 levels at the time of ADRs had eosinophilia.

CD4^+^ and CD8^+^ T cells in skin biopsies might have been recruited through MIG (21) and IP-10 (22) which are increased in subjects with ADRs. Notably, whereas MIG and IP-10 are produced by monocytes, IP-10 is also secreted by T lymphocytes (22) supporting the activation of the innate and adaptive immune responses in benznidazole-induced dermatitis. Since IP-10 and MIG are induced by IFN-γ, these chemokines are associated with a Th1 profile (23). IFN-γ-producing cells in response to benznidazole were detected in a few subjects 6 to 24 months after benznidazole treatment, supporting that at least in some cases, a Th1 response characteristic of type IVa might occur in benznidazole ADRs. The concomitant increase in serum levels of IL-5, soluble FAS-L, IP-10, and MIG supports a mixed Th1, Th2, and cytotoxic profile with the involvement of T cells, eosinophils, and macrophages. That T-bet (24, 25) is also involved in the differentiation of cytotoxic lymphocytes is supported by the many CD8^+^T cells, along with the expression of granzyme B and perforin in tissues expressing T-bet.

Other authors have reported that the lymphocyte transformation test detected benznidazole-reactive cells in subjects who experienced skin ADRs to benznidazole (11). Contrasting with the ELISPOT assays conducted in our study, the lymphocyte transformation test is based on a 6-day culture in which drug-specific T cells are expanded: thus, the sensitivity might be improved, although nonspecific activation of T cells can also occur. The mediators found to increase were previously associated with the clinical manifestations of ADRs to other drugs, including maculopapular rash and exanthema with or without eosinophilia (12, 26–28). A heterogeneous pattern of cytokines involving CD4^+^ and CD8^+^ T cells has been described in ADRs with the development of maculopapular exanthema (19, 29), and two cases of overlapping clinical manifestations of drug reaction with eosinophilia and systemic symptoms and Stevens-Johnson syndrome/toxic epidermal necrolysis have been reported in subjects infected with T. cruzi treated with benznidazole (30).

The main models proposed to explain the activation of the immune system by drugs comprise haptenization, which results in alteration of autologous proteins either directly or after metabolization, and the “pharmacological interaction of drugs with immune receptors” (P-I) model by which a drug can bind directly to the T cell receptor or certain human leukocyte antigen (HLA) molecules (7, 31). More recently, the “heterologous-immunity” model has been proposed, in which memory T cells specific for a prior infection may cross-react with endogenous peptides presented in the presence of a drug in the context of HLA (31). Although which of these models apply to benznidazole-induced hypersensitivity reactions cannot be ascertained, what is likely is that resident memory T cells in the skin of subjects infected with T. cruzi represent preactivated T cells; thus, a P-I model is plausible. The skin is a border region with a dense network of antigen-presenting cells and sentinel T cells, ready to mount an immune response (32, 33). We found that subjects with ADRs to benznidazole had a higher frequency of activated T cells before the initiation of treatment than those without ADRs and intraepidermal CD8^+^T cells colocalized with the extensive expression of the dendritic cell marker CD1a^+^. Drug-induced activation of local T cells in the skin by a P-I model is correlated with the concentration of the drug and facilitated by the close contact of T cells to dendritic cells in the skin that provide costimulation signals (7). Upon T cell stimulation, cytokine secretion is induced, providing a danger signal to local epidermal and endothelial cells (7). We have shown that the intermittent administration of benznidazole results in milder skin ADRs with a low rate of treatment suspension than with a daily dose administration (34, 35). A possibility is that an intermittent drug scheme avoids reaching the threshold for T cell activation and the induction of the ADRs. However, the high inflammatory response elicited during ADRs might also help clear the infection, accounting for the conversion to negative serological findings observed in a proportion of the subjects with incomplete treatment schedules with benznidazole due to ADRs (36, 37).

The baseline LDL level appeared to be an independent predictive marker of ADRs. Dyslipidemia, commonly presented as hypercholesterolemia and hypertriglyceridemia, has been reported in subjects with chronic Chagas disease (38). Increased LDL might promote its modification by oxidation and aggregation, and this modified LDL might function as a ligand for macrophage pattern recognition receptors that directly trigger proinflammatory signaling (39). Additionally, modified LDL-treated dendritic cells have been reported to elicit strong Th1 reaction and T cell proliferation (40). Uremia levels were also associated with ADRs, an observation that might account for the increased production of inflammatory cytokines, reactive oxygen species, and activation of proinflammatory subsets of T cells and monocytes observed in uremic subjects (41). Therefore, chronic immune activation in subjects infected with T. cruzi might be a risk factor for ADRs.

Dermatitis was also associated with high GOT levels. A dysfunctional liver metabolism might inhibit the bio-inactivating process, which might abolish the toxic and immunogenic properties of benznidazole metabolites activated in the liver by cytochrome P450 enzymes (42). In contrast with other studies (43), we did not find differences in the rate of appearance of side effects among males and females. Other authors have also claimed that adverse benznidazole reactions may be genetically determined, but very strong associations have not been found (44–46).

Overall, our findings identified baseline risk factors associated with the development of ADRs to benznidazole and identified a set of serum cytokines and chemokines whose levels might eventually predict the appearance of ADRs during treatment with benznidazole.

### Limitations of the study.

We cannot rule out that benznidazole-derived metabolites are the target antigens of the hypersensitivity reactions, and thus the use of the native drug might account for the low responses in the in vitro ELISPOT asssays. The proportion of males and females recruited was not the same; an issue that can be explained somehow by a higher rate of acceptance to participate in research protocols among women than men. Because of the complexity of the assays and biopsy collection, the sample size of the study population was relatively low.

## MATERIALS AND METHODS

### Selection of study population.

A total of 59 subjects with confirmed diagnosis for chronic Chagas disease were recruited in the Chagas Disease Unit of Hospital Eva Perón, Buenos Aires, Argentina. T. cruzi infection was determined using indirect immunofluorescence assay, hemagglutination, and enzyme-linked immunosorbent assay (ELISA) techniques (1) (*n* = 59 subjects). Physical evaluation, electrocardiography, and echocardiography were performed on all subjects and stratified according to a modified clinical classification of Kuschnir (34): T. cruzi-infected subjects with indication of etiological treatment with benznidazole were invited to participate in this study. Those who accepted were consecutively included and monitored during a follow-up period of two years. Subjects who experienced dermatitis were assigned to the group of patients with benznidazole-induced dermatitis and those who completed the treatment scheme without the appearance of dermatitis were assigned to the group without cutaneous reactions. Patients who showed other adverse events during benznidazole administration not involving skin reactions were excluded from the protocol. Besides, individuals with ischemic heart disease, cancer, human immunodeficiency virus infection, syphilis, diabetes, arthritis, or serious allergies were excluded from the study Benznidazole was administered at a dose of 5 mg/kg/day for 30 days. ADRs were considered mild when localized skin rash and pruritus were evident, which generally subsided without medication. Moderate and severe dermatitis comprised a larger area of skin rash compared with mild ADRs and was generally associated with fever, arthralgia, and vomiting (47) (Table 1). The uninfected control group consisted of 24 age-matched subjects of areas where T. cruzi infection was not endemic who were found to be negative for T. cruzi by serologic testing (Table 1). This study was approved by the institutional ethics committee. Signed informed consent was obtained from all individuals prior to inclusion in the study.

### Collection of peripheral blood mononuclear cells and serum specimens.

Fifty milliliters of blood were drawn from each subject via venipuncture using heparinized Vacutainer tubes (BD Biosciences) before treatment, at the time of the adverse effect, and thereafter at 3, 6, 12, and 24 months after the initiation of treatment and PBMCs were isolated using FIcoll-hypaque (GE) density gradient centrifugation and were cryopreserved for later analysis. Ten milliliters of blood from each subject was coagulated at room temperature and centrifuged at 1,000 × *g* for 15 minutes for serum separation. Assays were not performed for all samples, owing to the limited quantity of blood samples.

### Benznidazole.

The benznidazole (Sigma-Aldrich) drug was dissolved in dimethyl sulfoxide (DMSO) and diluted in RPMI 1640 medium supplemented with FBS 10% media up to the final concentration required for each experiment. A mashed preparation of the commercial benznidazole product Radanil (Roche) dissolved in DMSO was also assessed.

### Determination of benznidazole cytotoxic activity on viable cells.

One million PBMCs per milliliter were stimulated with 10 to 400 μg/mL of benznidazole or 0.4% of the diluent DMSO alone for 20 to 72 h at 37°C and 5% CO_2_. At the end of the incubation, the frequency of dead cells was measured by staining with 7-aminoactinomycin D (7AAD; BD Biosciences, San Jose, CA, USA) and the monoclonal antibodies anti-CD4 FITC (555346; BD) and CD8 PE (555367; BD) for 30 min at 4°C. Samples were acquired using a FACS Calibur cytometer (BD) and analyzed using FlowJo software version 10 (TreeStar, San Carlos, CA, USA).

### Interferon-γ, interleukin-2, and IL-5 enzyme-linked immunosorbent spot assays.

The number of benznidazole-responsive IFN-γ-, IL-2-, and IL-5-secreting T cells was determined by *ex vivo* ELISPOT assays, using a commercial kit (ELISPOT Human IFN-γ or IL-2 or IL-5 ELISPOT Set; BD Biosciences), as described elsewhere (48) Responses were considered positive when (i) a minimum of 10 spots/4 × 10^5^ PBMCs were present per well, and (ii) the number of spots was at least twice the value of that of the wells stimulated with DMSO alone (49).

### Cytometric bead array assays.

Cytometric bead array (CBA) assays were conducted according to the manufacturer’s instructions (BD Biosciences) to determine the presence of IL-5, IL-10, IL-13, eotaxin, granzyme B, and chemokines (i.e., IP-10, MCP-1; MIG, IL-8, and RANTES) in serum samples of subjects infected with T. cruzi and those who were uninfected. The samples were acquired on a FACS Aria II flow cytometer, and the data were analyzed by using BD FCAP Array Software, version 1.4 (BD Biosciences).

### ELISAs for soluble Fas cell surface death receptor ligand.

Serum samples were analyzed for the presence of soluble FAS-L by ELISA, using a commercial kit (catalog no. ab100515; ABCAM) according to the manufacturer’s instructions.

### Evaluation of CD69 expression in CD4^+^ and CD8^+^ T cells.

One million PBMCs were stained with anti-CD3 APC (340440; BD), anti-CD4 FITC (555346; BD), anti-CD8 PerCP (BD; 347314), and anti-CD69 PE (BD; 555531) for 30 min at 4°C. Samples were acquired using an FACSCalibur cytometer (BD) and analyzed using FlowJo software version 10 (TreeStar).

### Immunohistochemical analysis of skin biopsies.

Skin biopsies were obtained from patients with severe dermatitis caused by benznidazole therapy. Skin samples were fixed in 4% buffered formalin, and paraffin-embedded sections of 3-μm thickness were stained with hematoxylin and eosin.

Immunohistochemical staining was performed using the BenchMark GX System (Roche, Basel, Switzerland), according to the standard protocols supplied by the manufacturer. After deparaffinization, the tissue sections were treated with cell conditioning reagent 1 (CC1; Roche no 950-124) for antigen retrieval. The Optiview DAB IHC Detection Kit (Roche no. 760-700) was used for visualization. A list of the antibodies assessed is shown in Supplementary Table S1. The samples were analyzed using a Zeiss Axiophot optical microscope.

### Measurement of biochemical and hematologic markers.

Routine blood tests (Table 2) were performed prior starting benznidazole treatment using the CELL-DYN Ruby Hematology Analyzer (Abbott, Chicago, IL, USA).

### Statistics.

The normality of the distribution of variables was assessed using the Kolmogorov–Smirnov criterion, and the groups were compared using the unpaired *t* test or the Mann-Whitney *U* test, as appropriate. Univariate analysis was performed using the Wilcoxon rank sum test and the two-sample *t* test for continuous variables and the Fisher exact test for categorical variables. Logistic regression analysis was performed with the variables given *P* ≤ 0.05 by the Univariate analysis. Differences were considered statistically significant at *P* < 0.05. Statistical analyses were conducted using GraphPad Prism v9.0 and Statistix 10 software (Analytical Software, Tallahassee, TN, USA).
