# Supplementary material for: Mixed T Helper1/T Helper2/T Cytotoxic Profile in Subjects with Chronic Chagas Disease with Hypersensitivity Reactions to Benznidazole
Source: Microbiol Spectr. 2022 Aug 8;10(4):e01357-22. doi: 10.1128/spectrum.01357-22 (PMC9430713; doi:10.1128/spectrum.01357-22)
Supplement: Supplemental file 1 — Supplemental material. Download spectrum.01357-22-s0001.pdf, PDF file, 0.3 MB [file spectrum.01357-22-s0001.pdf]

Supplementary Table I

Anti-human	Fluorochrome [A]	Clone	Brand
CD1a	Purified	EP3622	Roche-Ventana
CD3	APC	SK7	BD Biosciences
CD4	FITC	RPA-T4	BD Pharmingen
CD4	Purified	SP35	Roche-Ventana
CD8	PerCP	SK1	BD Biosciences
CD8	Purified	SP57	Roche-Ventana
CD69	PE	FN 50	BD
Granzyme B	Purified	Polyclonal	Roche-Ventana
GATA-3	Purified	L50-823	Roche-Ventana
IFN-γ	Purified	MD-1	Biolegend
IL-2	Purified	Component 51-2516KZ	BD Biosciences
IL-5	Purified	TRFK5	Biolegend
Perforin	Purified	dG9	Abcam
T-bet	Purified	4B10	BD Pharmingen

Notes. [A] Allophycocyanin (APC), peridinin chlorophyll (PerCP), fluorescein isothiocyanate (FITC), phycoerythrin (PE). NA, not available.

Supplementary Figure 1

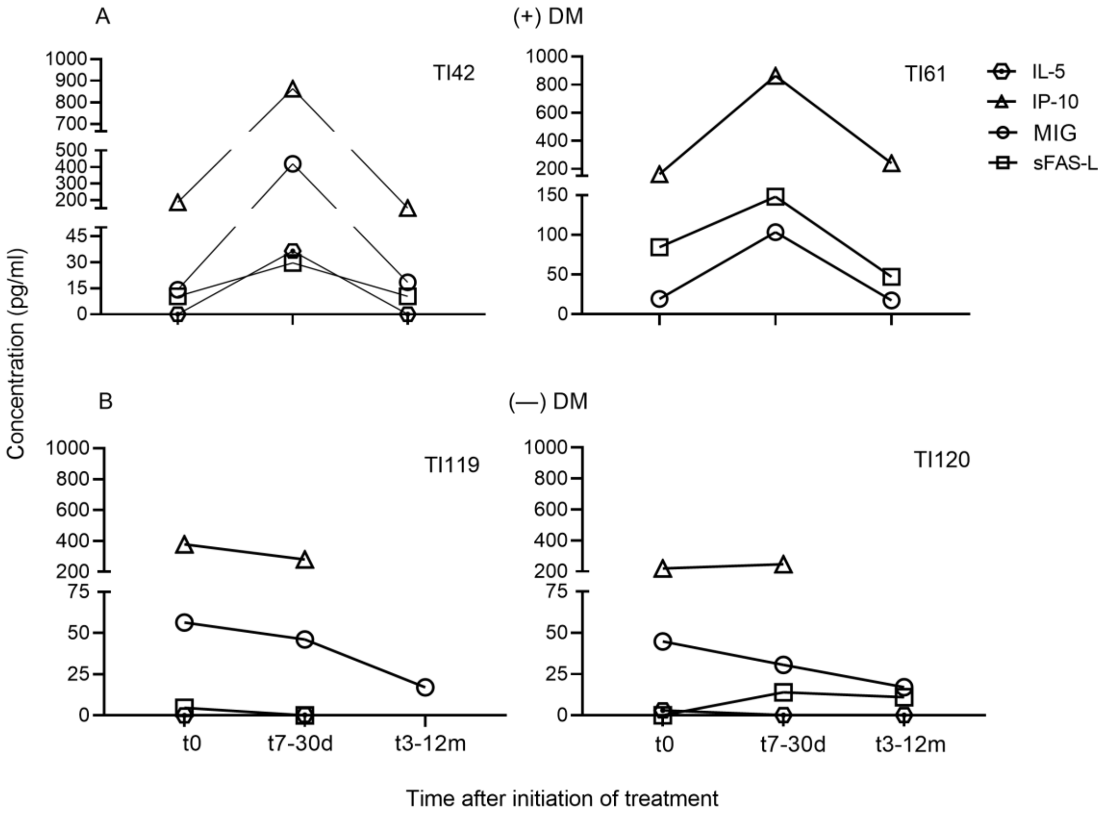

Supplementary FIG 1. Mixed profiles of cytokines and chemokines in the sera of patients with Chagas disease with adverse skin reactions during treatment with benznidazole. Serum samples were taken prior to treatment (t0), at 7-30 days (t17-30d) and at 3-12 months (t3-12m) after initiation of treatment in *T. cruzi*-infected subjects with moderate/severe dermatitis [A, (+) DM] or without skin reactions [B, (─) DM] during benznidazole therapy. Plots exhibit representative data for single subjects with (TI42 and TI61) or without (TI119 and TI120) ADRs.

Supplementary Figure 2

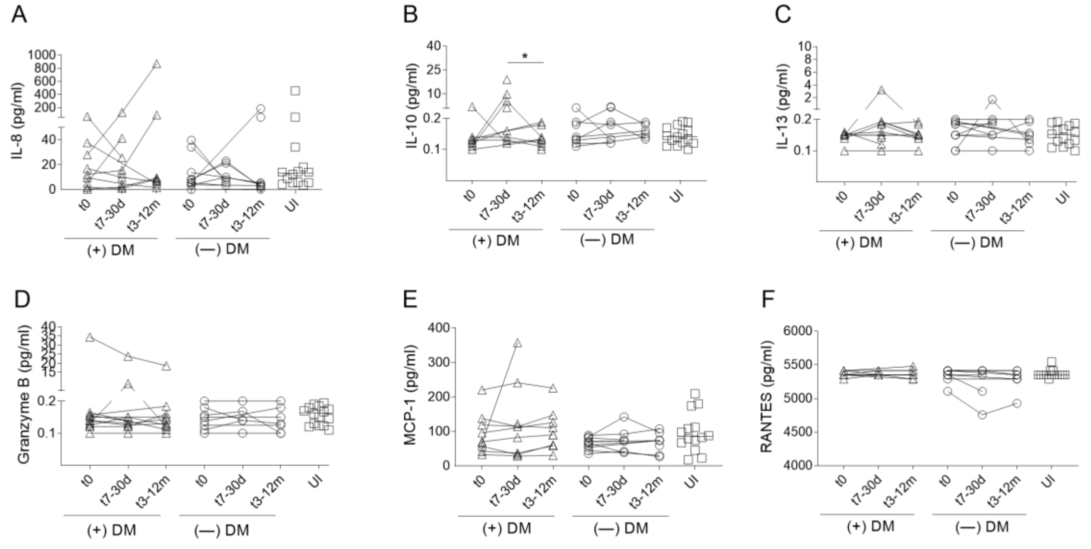

Supplementary FIG 2. CBA assays for the measurement of cytokines and chemokines in the sera of patients with Chagas disease treated with benznidazole. Serum samples were taken prior to treatment (t0), at 7-30 days (t17-30d) and 3-12 months (t3-12m) after initiation of treatment in *T. cruzi*-infected subjects with moderate/severe (+ DM; n= 11) or without (— DM, n= 11) skin reactions during benznidazole therapy and in the uninfected subjects (UI, n=14). Each point represents the concentration values for a single subject for each soluble factor evaluated: granzyme B (A), IL-13 (B), MCP-1 (C), RANTES (D) and IL-8 (E). No differences were recorded among time points posttreatment or between *T. cruzi*-infected and uninfected groups.
